# Sustainable Development Advantages of Cross-Laminated Timber (CLT) and Cross-Laminated Bamboo and Timber (CLBT)

**DOI:** 10.3390/ma18214913

**Published:** 2025-10-27

**Authors:** Jinping Li, Kang Zhao

**Affiliations:** 1 Office of Science and Technology, Jinling Institute of Technology, Nanjing 211199, China; ljp@jit.edu.cn; 2 College of Civil Engineering, Nanjing Forestry University, Nanjing 210037, China; 3 Jiangsu Carbon Sequestration Materials and Structural Technology of Bamboo & Wood Research Center, Nanjing Forestry University, Nanjing 210037, China

**Keywords:** bio-based materials, construction materials, engineered bamboo, structural form, structural performance

## Abstract

As an innovative advancement beyond cross-laminated timber (CLT), cross-laminated bamboo and timber (CLBT) combines sustainability with enhanced structural performance. This review critically assesses the current state of CLBT research, focusing on its failure mechanisms, mechanical properties, and predictive theoretical models. Key findings indicate that CLBT exhibits superior rolling shear strength, bending stiffness, and stability compared to conventional CLT, achieved through optimized hybrid layering and manufacturing techniques. The integration of bamboo not only improves mechanical performance but also promotes diversification of raw materials and more efficient use of regional biomass. This paper highlights the potential of CLBT as a high-performance, eco-friendly construction material and identifies key research gaps and future directions to facilitate its standardized application.

## 1. Introduction

Timber construction, as a traditional form of building, has a long history and is found worldwide. Using timber as the primary building material offers advantages such as low carbon emissions, environmental friendliness, renewability, and low processing energy consumption. However, due to the limitations of the mechanical properties, dimensional stability, and fire and corrosion resistance of natural timber, traditional timber structures are mainly used for low-rise residential buildings, garden architecture, and temporary structures. They are not suitable for meeting the demands of modern architecture for high-rise, large-span, and complex-functional buildings [1,2,3,4].

To overcome the limitations of solid timber materials, various engineered timber products have emerged. Among them, cross-laminated timber (CLT), as a representative of innovative engineered timber materials, has gained international recognition since its introduction in Europe in the 1970s due to its excellent mechanical properties and construction convenience. CLT is made by gluing multiple layers of sawn timber or structural panels in orthogonal orientations, providing good in-plane stiffness and bidirectional load-bearing capacity. It can be used for various load-bearing components such as walls, floors, and shells, greatly expanding the application range of timber structures [5,6,7]. From multi-story timber school buildings in Northern Europe to the 18-story student dormitory Brock Commons in North America, CLT has become an important material driving the development of modern timber structures for high-rise and large-span applications.

Currently, relevant standards such as EN 16351:2021 Timber structures—Cross laminated timber (EN 16351) in Europe and ANSI/APA PRG 320-2019: Standard for Performance Rated Cross-Laminated Timber (ANSI/APA PRG 320) in North America have gradually been established, providing technical support for the standardized design, production, and application of CLT. Although CLT technology has become increasingly mature, its further development still faces challenges, such as limitations in raw material sourcing and performance bottlenecks. On the one hand, traditional CLT primarily relies on softwood, such as spruce and fir, with the resource supply becoming increasingly scarce. On the other hand, the weak transverse layer rolling shear performance limits the application of CLT in large-span heavy-load structures. To address these issues, researchers have started exploring hybrid CLT by introducing broadleaf timber, bamboo, or engineered timber panels to enhance the performance of the transverse layers, thus improving the material’s mechanical properties and broadening the range of available raw materials [8,9,10,11,12].

Meanwhile, bamboo, as a rapidly growing biomass material with excellent mechanical properties, has attracted widespread attention in the engineering field. As a major bamboo-producing country, China has a natural advantage in the development of bamboo structures. However, natural bamboo faces challenges in direct application to building structures due to structural defects such as anisotropy, dimensional instability, susceptibility to cracking, and poor durability [13,14]. Especially when subjected to conditions that allow the development of rotting fungi and bamboo borers (*Dinoderus minutus*). To overcome the inherent limitations of natural bamboo, various modification techniques have been developed. On one hand, thermal treatment has been proven to significantly enhance the physical properties and durability of bamboo [15,16]. On the other hand, engineered bamboo materials (such as bamboo scrimber [17,18], laminated bamboo [19,20], Glubam [21,22], and inorganic-bonded bamboo [23,24]) produced through processes like recomposition, gluing, and lamination have effectively improved the anisotropic nature and susceptibility to fungal decay of natural bamboo. These materials exhibit tensile and compressive strengths that far exceed those of ordinary timber, making them suitable for load-bearing components such as beams, columns, and slabs. In recent years, research on engineered bamboo structures has developed rapidly, with significant progress in basic material mechanical properties [13,14,15,16,17,18,19,20], bamboo–steel composite [25,26,27,28,29,30], bamboo–concrete composite [31,32,33,34,35], and the seismic performance [36,37,38] of overall structures. These advancements have laid a solid foundation for the large-scale application of engineered bamboo in construction.

Based on the mature experience of CLT and the high-performance characteristics of engineered bamboo materials, the combination of bamboo and timber into CLBT has gradually emerged as a new research direction [39]. CLBT, achieved by cross-laminating bamboo with timber, not only inherits the advantages of CLT but also integrates the high strength of bamboo, thereby improving the load-bearing efficiency and ductility of components. Additionally, CLBT contributes to the high-value utilization of bamboo, effectively reducing the overreliance on forest resources. Currently, research on CLBT is still in its early stages, covering areas such as the static performance, failure modes, and theoretical models of bamboo–timber composite beams and columns, and has demonstrated superior mechanical potential compared to traditional CLT [40,41,42,43,44]. However, there remain significant gaps in design methods, connection details, long-term performance, and standardization. This paper provides a comprehensive synthesis of the state-of-the-art research on CLT and CLBT. It examines their development across multiple domains—including material processing, structural performance under various loads, failure mechanisms, and design methodologies for fire and earthquakes—with the ultimate goal of guiding their optimized application and fostering technological advances in green building practices.

## 2. CLT Structure

### 2.1. Development Process

**Figure 1:**
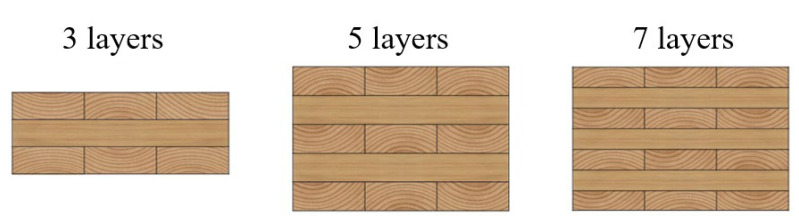
Cross-section of common CLT board units.

With the advancement of industrial technology and the growing demand for environmentally friendly construction, multi-story and even high-rise timber buildings have gradually emerged. Among them, CLT, as a representative engineered timber product, has been widely used worldwide for structural components such as roofs, floors, and walls since it was first proposed in Europe in the 1970s [45,46,47,48,49,50]. CLT is a large-format engineered timber product manufactured through a cross-laminated gluing process. A typical CLT panel consists of multiple layers (commonly three, five, or seven) of solid-sawn lumber (Figure 1), with adjacent layers arranged orthogonally at 90° and bonded into an integral panel using structural adhesives under high pressure [51]. This cross-laminated configuration imparts CLT with unique bidirectional mechanical properties: the layers aligned with the grain (0° layers) provide high tensile and compressive strength, while the layers perpendicular to the grain (90° layers) effectively resist shear stresses, enabling CLT to serve as a substitute for concrete or steel in load-bearing walls, floors, and roof panels.

**Figure 2:**
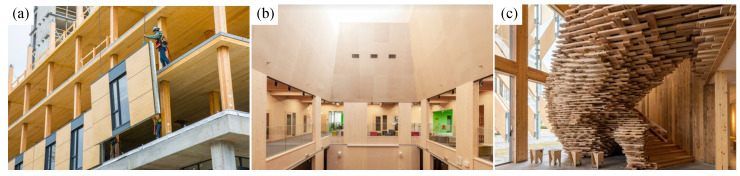
Architectural applications for CLT structures. (**a**) Brock Commons Talltimber House [52] (**b**) Kuhmo Timber School [53]; (**c**) CLT Park Harumi [54].

With the growing demand for low-carbon, eco-friendly, and sustainable living environments, CLT components are being increasingly applied in modern timber construction, ranging from small- and medium-scale demonstration projects to large public buildings. Compared with traditional building materials, CLT offers numerous advantages (such as design flexibility, environmental sustainability, factory prefabrication, and light weight), which give it broad application prospects in both single-story and multi-story residential buildings [51]. As shown in Figure 2a, the Brock Commons Talltimber House on the University of British Columbia Campus in Vancouver is an 18-story CLT-concrete student residence. The first floor is constructed with concrete, while the remaining 17 stories are timber, supported laterally by two central reinforced-concrete cores. Both the walls and floor slabs of the building are made of prefabricated CLT elements. The entire superstructure was completed within 70 days, approximately four months ahead of schedule compared with similar projects. As shown in Figure 2b, the Kuhmo School of Timber in Finland is a CLT structure in which the main frame, walls, and floor slabs are all constructed from CLT, reducing carbon emissions while creating a warm and natural learning environment. As shown in Figure 2c, CLT Park Harumi in Tokyo, Japan, is a temporary building complex constructed entirely with CLT. The project emphasizes material reusability, as the buildings can be completely disassembled after use and reconstructed at other sites, fully demonstrating the circular economy potential of CLT structures.

### 2.2. Research Progress on CLT

#### 2.2.1. Failure Modes

Under high stress, the failure mechanism of CLT differs from that of traditional timber structures due to the presence of one or more cross layers whose grain direction is perpendicular to that of the outer layers. Because timber exhibits low tensile strength perpendicular to the grain, these cross layers are prone to brittle failures, such as cross-grain splitting, which can ultimately lead to the failure of the entire structural system. Common failure modes of CLT include tensile failure in the outer tension layer and shear damage in the cross layers. When CLT components are subjected to external loads, rolling shear stresses can readily develop between layers, leading to interlayer fractures. Such failures often occur in floor slabs or wall panels during long-term service and are difficult to detect visually, thereby posing significant risks to structural safety.

**Figure 3:**
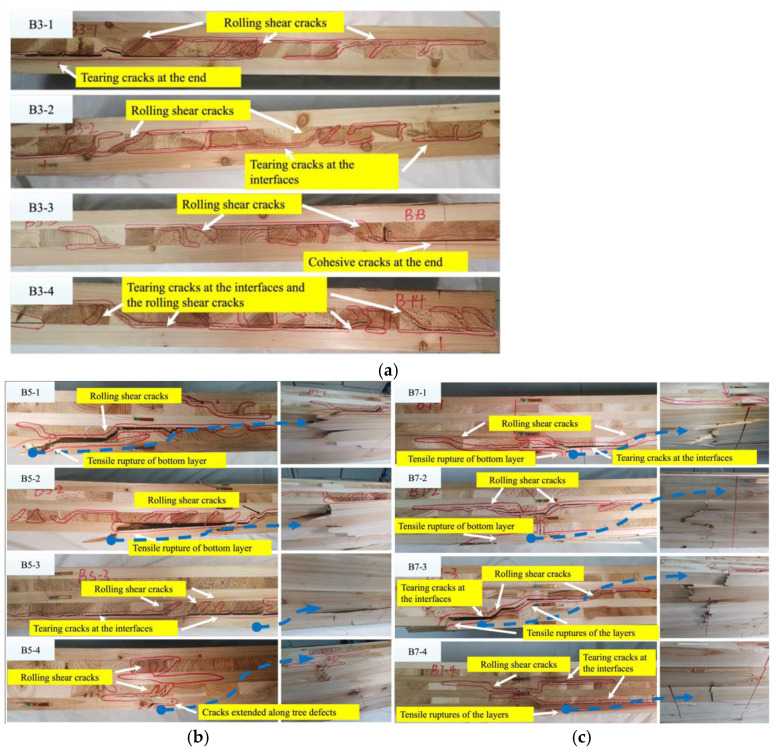
Failure modes of CLT [55]. (**a**) 3-layer CLT. (**b**) 5-layer CLT. (**c**) 7-layer CLT.

**Figure 4:**
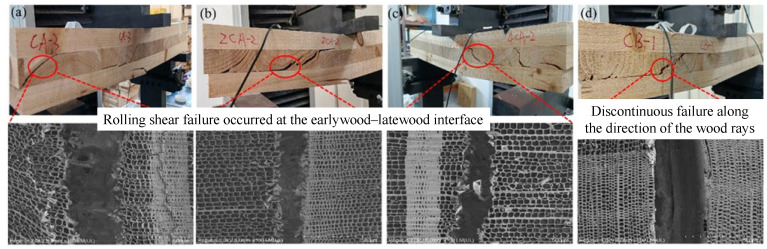
Interlayer shear failure modes of CLT and SEM images [55]. (**a**–**c**) Rolling shear failure occuired at the earlywood-latewood interface. (**d**) Discontinuous failure along the direction of the wood rays.

As shown in Figure 3, Kong et al. [55] found that CLT with different layer configurations exhibit significantly different failure modes under bending tests: three-layer specimens primarily experienced rolling shear and interlayer delamination in the cross layers, with no tensile rupture observed in the bottom layer; five-layer and seven-layer specimens predominantly failed due to tensile rupture of the bottom lamella, accompanied by a certain degree of rolling shear cracking within the cross layers. As shown in Figure 4, Qu et al. [56] analyzed rolling shear failure from the perspective of timber cellular structure using scanning electron microscopy (SEM) and reported that such damage typically occurs at the interface between early-timber and late-timber, manifesting as discontinuous cracks propagating along the timber rays. This is attributed to the lower strength of early-timber cells compared with late-timber cells, causing shear failure to initiate within or near early-timber regions.

Numerous scholars have investigated the failure modes of CLT in bending strength tests, including Wang et al. [57], Sikora et al. [51], Buck et al. [58], Niederwestberg et al. [59], Norwahyuni et al. [60], and Alia Syahirah et al. [61]. Their studies generally indicate that CLT specimens under bending primarily exhibit three typical failure modes, with some specimens showing two or even all three failure mechanisms simultaneously. The main failure types include: (1) tensile failure of the outermost tension layer due to timber defects; (2) rolling shear failure occurring near glue lines, accompanied by shear strain perpendicular to the grain; and (3) longitudinal shear failure along the grain direction. It is worth noting that Niederwestberg et al. [59] reported that introducing laminated strand lumber (LSL) into the cross layers can effectively mitigate the rolling shear failure commonly observed in CLT. In addition, Hochreiner et al. [62] examined various failure modes of CLT panels under concentrated loads through bending tests, focusing on the internal structural responses associated with different failure mechanisms and providing in-depth insights into the underlying failure processes.

#### 2.2.2. Mechanical Properties

Rolling shear performance plays a critical role in the design of CLT products, particularly when they are used as floor or roof panels subjected to substantial vertical loads. Due to the presence of cross-laminated layers within CLT, loads applied perpendicular to the panel surface tend to induce rolling shear stresses, which can damage the cross-layers and ultimately lead to structural failure. The rolling shear stiffness and strength of CLT are key parameters governing its load-bearing capacity and overall performance in floor and roof applications. Current research focuses on the factors influencing the rolling shear behavior of CLT, which can be broadly categorized into two aspects: (1) variations in the types of lamination materials, including softwood, hardwood, and various wood-based composites; (2) differences in manufacturing processes, such as layup configurations, adhesive bonding techniques, and lamella thickness.

##### Lamination Materials

Currently, CLT production primarily utilizes softwood such as spruce-pine-fir (SPF). However, the relatively low rolling shear modulus and strength of softwood limit its application in scenarios demanding high shear performance, such as high-rise buildings or large-span structures. For example, the rolling shear strength of CLT fabricated from SPF is typically only about 1.5 MPa [63], which is insufficient to meet the load-bearing demands of heavily loaded floors or shear walls. To overcome this performance bottleneck, researchers have increasingly explored the development of hybrid CLT (HCLT). By incorporating superior-performing layer materials (such as timber from different tree species, bamboo, or timber-based composite panels), HCLT not only significantly enhances shear performance but also broadens raw material sources, optimizes mechanical properties, and helps reduce manufacturing costs [64].

Aicher et al. [65] investigated the feasibility of incorporating *Fagus sylvatica* into the cross-layers of CLT, and the results showed that the rolling shear strength and modulus of beech were approximately five and seven times higher than those of softwood, respectively. Furthermore, Aicher et al. [66] evaluated the out-of-plane bending behavior of HCLT panels composed of *Picea abies* in the longitudinal layers and European beech in the cross-layers through four-point bending tests. Their findings demonstrated that such HCLT panels exhibit excellent structural performance and hold significant potential for applications in building construction. Gong et al. [67] reported that the rolling shear strength of Populus alba and Betula pendula was 2.8 MPa and 3.1 MPa, respectively, which is substantially higher than that of spruce (0.5 MPa). In addition, their failure modes tended to exhibit ductile shear behavior rather than brittle fracture, a feature that is particularly beneficial for seismic design. Hematabadi et al. [10] investigated the bending performance of three-layer HCLT panels composed of *Populus alba* as longitudinal layers and *Fagus orientalis* as cross-layers. The results indicated that hybrid-species HCLT outperformed single-species CLT in terms of bending behavior. Sciomenta et al. [68] conducted both in-plane and out-of-plane tests on HCLT fabricated from *Fagus orientalis* and *Pinus nigra* subsp. *laricio*, and both experimental and numerical results confirmed its superior performance under bending and shear loads. In addition, studies on the shear performance, bonding performance, and fatigue performance of HCLT composed of lumber and oriented strand board (OSB) have all confirmed its superiority over conventional CLT [9,11,69].

In summary, manufacturing HCLT by combining different timber species or engineered bamboo–timber products with solid lumber can diversify the sources of lamina materials and reduce the consumption of solid sawn timber; compared with conventional CLT, well-designed HCLT can achieve superior physical and mechanical properties.

##### Manufacturing Processes

The bonding performance has a significant impact on the structural quality, stability, and in-service performance of CLT in practical applications. Gong et al. [67] investigated the bonding process of fast-growing poplar CLT and found that using a one-component polyurethane adhesive yielded the best results, with the optimal manufacturing parameters being: adhesive spread of 180 g/m^2^ and pressing pressure of 1.0 MPa. Li et al. [70] reported that CLT specimens made from SPF under a pressing pressure of 0.4 MPa achieved a rolling shear strength of 2.22 MPa, which was markedly higher than the 1.85 MPa strength of similar specimens produced under 0.1 MPa. Yusof et al. [60] observed that CLT specimens bonded with phenol-resorcinol-formaldehyde (PRF) exhibited a lower delamination rate compared to those bonded with polyurethane (PUR), though both adhesives met the delamination resistance requirements specified in Timber structures—Cross laminated timber—Requirements (EN 16351:2015). Research by Santos et al. [71] demonstrated that increasing pressing pressure reduces the delamination rate of CLT and that pre-treatment of the bonding layer can significantly enhance bonding quality.

The thickness of the lamella is an important dimensional parameter of CLT. Numerous studies have consistently shown that the lamella thickness significantly affects the mechanical properties of CLT, with rolling shear strength decreasing as the lamella thickness increases [51,72]. The fire resistance of CLT is also influenced by lamella thickness. Experimental results [73,74] have shown that, under the same total thickness, five-layer CLT experiences char layer detachment earlier than three-layer CLT. This is because the outer layers of CLT are thicker, and their char layer is less likely to peel off during combustion, resulting in better fire resistance. The mechanical properties of CLT are not only affected by the thickness of the lamella but also by the performance differences caused by changes in the width-to-thickness ratio of the lamella. Studies have indicated [75,76] that as the width-to-thickness ratio decreases, CLT is more prone to rolling shear failure, and the width-to-thickness ratio has a significant effect on the rolling shear modulus, showing a positive correlation. Additionally, the impact of changes in the width-to-thickness ratio on the overall stiffness of CLT is also noteworthy. Turesson et al. [77] studied the effect of the width-to-thickness ratio of the lamella on the in-plane shear stiffness of three-layer CLT. The results showed that, for a fixed total thickness, increasing the width-to-thickness ratio of the odd-numbered layers improved the in-plane shear stiffness of CLT. Berg et al. [78] used finite element analysis to simulate the mechanical performance of CLT under uniformly distributed out-of-plane loads with different lamella width-to-thickness ratios. Their results indicated that as the width-to-thickness ratio increased, the overall stiffness of CLT also increased.

**Figure 5:**
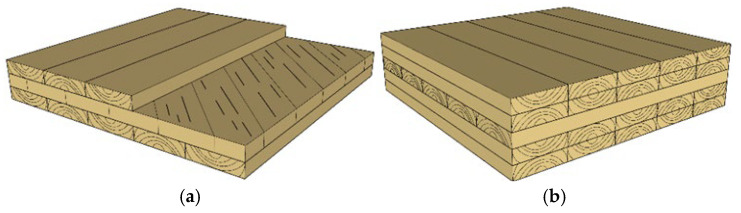
Lay-up structural design of CLT. (**a**) Diagonal assembly (45°). (**b**) Outermost two layers parallel to grain.

In addition, the layup angle between the layers of sawn timber also influences the mechanical properties of CLT. CLT is typically manufactured with an orthogonal layup, but this configuration may result in reduced interlayer shear performance compared to engineered timber products such as laminated timber [79]. To improve the mechanical properties of CLT, its layup structure can be designed, such as using a 45° layup for the cross layers (Figure 5a) or a continuous two-layer parallel layup (Figure 5b). Dietrich et al. [58] studied the bending performance of CLT with 45° and 90° layups of the cross layers, finding that the bending strength of the former was 35% higher than that of the latter. However, CLT with a 45° layup could not fully overcome the damage caused by rolling shear stresses in the cross layers under external loads. Later, Dietrich et al. [80] compared the compressive performance of these two types of CLT through in-plane compression tests and found that CLT with a 45° layup exhibited better compressive performance, with compressive stiffness and strength being 30% and 15% higher, respectively, than those of the 90° layup CLT. Matthias et al. [81] investigated the effect of cross-layer orientation (30°, ±45°, or 60°) on the mechanical properties of diagonal laminated timber (DLT). Compared to CLT, DLT showed a significant improvement in torsional stiffness, with its main advantage being in uniaxial load transfer. In floor systems under normal serviceability limit states, DLT is more likely to meet performance requirements. Due to the 45° diagonal layup, the intermediate layer is subjected to forces between those acting perpendicular and parallel to the grain, and compared to the cross layers in an orthogonal layup, this layer can resist shear stresses, resulting in increased compressive elastic modulus and compressive strength. These studies all confirm that optimization of the manufacturing process significantly improves the mechanical properties of CLT.

##### Fire and Seismic Resistance

The mechanical behavior of CLT under extreme environmental conditions, particularly fire and earthquakes, is critically important for ensuring the safety and reliability of modern timber structures. As a result, this area has attracted significant research attention in recent years. Studies have focused on understanding charring behavior, load-bearing capacity retention, and fall-off resistance of CLT panels under high temperatures, as well as their hysteresis performance, energy dissipation capacity, and connection integrity under cyclic seismic loads. These investigations are essential for developing robust design guidelines and advancing the adoption of CLT in regions with high seismic activity or stringent fire safety requirements.

**Figure 6:**
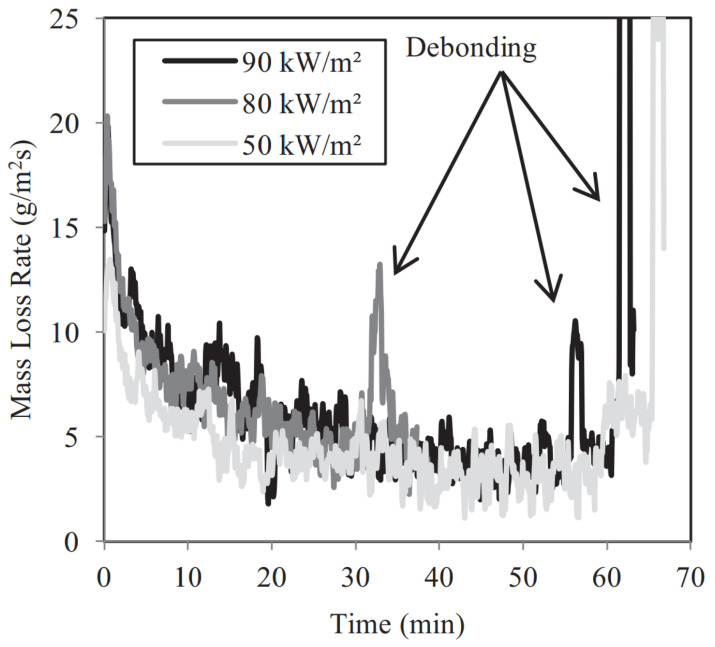
Effect of debonding on CLT mass loss rate at elevated temperature [83].

The behavior of solid timber in fire has been extensively studied, but the addition of adhesives in CLT introduces new challenges. In a fire, as charring progresses and heat waves approach the adhesive layers, high temperatures can degrade the adhesive properties, potentially leading to delamination of the CLT. Once delamination occurs, the charred layers no longer serve as thermal insulators, allowing oxygen to reach the uncharred lamellae [82]. McGregor et al. [83] conducted compartment fire tests on CLT and found that, in the absence of fire protection measures, the fire load, fire growth rate, and heat release rate of CLT significantly increased. When charring reaches the adhesive layers of CLT, polyurethane adhesives fail, resulting in delamination. Figure 6 illustrates that delamination exposes fresh timber to the fire, resulting in a sharp increase in mass loss at high temperatures. Wiesner et al. [84] studied the bending performance of CLT beams at high temperatures and found that the type of adhesive significantly influences the degradation of CLT beam stiffness. CLT made with PUR adhesive showed larger strain in the adhesive layer. Bateman et al. [85] conducted combustion tests on CLT and recommended that design standards should consider the energy balance between exposed timber surfaces and the smoke layer.

In seismic design, Azumi et al. [86] proposed a new spring system for connecting CLT, and experiments showed that a five-story CLT building using this system exhibited sufficient seismic resistance. Chen et al. [87] conducted full-scale shaking table tests on a CLT structure with dissipative diagonal braces and soft steel-rubber bearings, and the results demonstrated that the structure met the performance goals for dissipative connection replaceability and structural reparability under a rare 9-degree earthquake. Aloisio et al. [88] analyzed the seismic vulnerability of multi-story CLT structures and carried out a seven-story full-scale CLT structure earthquake simulation on the e-defense large-scale shaking table in Japan [89]. The test results indicated that the CLT panel structure exhibited excellent seismic integrity. Pei et al. [90] provided a detailed literature review on the application progress of CLT in seismic regions and offered design recommendations for multi-story CLT structures in high seismic zones.

#### 2.2.3. Theoretical Basis

In regions such as Europe and North America, the theoretical research and application of CLT are at the forefront, with the depth and breadth of related studies representing the advanced level in this field. In terms of theoretical calculations and simulations, Stürzenbecher et al. [91] studied and compared CLT panels with different layer combinations and pointed out that Ren’s composite plate theory is more suitable for the accurate and efficient calculation of CLT panels, particularly for the direct calculation of shear stress. Subsequently, based on Lekhnitskii beam theory and Ren’s plate theory, a more precise and efficient calculation method was proposed [92]. Flores et al. [93] conducted a multi-scale study and analyzed rolling shear failure in CLT structures using homogenization and cohesive zone models. Their constitutive model incorporated information about the cell walls, timber fibers, and growth rings, and a cohesive zone model was used on the macroscopic scale to simulate material cracks. Finally, the finite element calculation problem of CLT structures was solved through a hybrid domain decomposition strategy and parallel computing.

Similarly to modern timber structures, the connection performance of CLT is crucial. Vessby et al. [94] studied the structural characteristics of five-layer CLT panels and recommended specific connection methods to ensure the strength and stiffness of the connections. Schneider et al. [95] assessed the structural performance of connections between steel and CLT using American Society for Testing and Materials—ASTM standard methods and energy accumulation-based testing methods. Additionally, self-tapping screw reinforcement can effectively improve the overall load-bearing performance of timber-based composite structural elements, which is essential for achieving the curved shapes and long-span requirements of CLT materials [96,97].

## 3. Development of Modern Bamboo Structures

**Figure 7:**
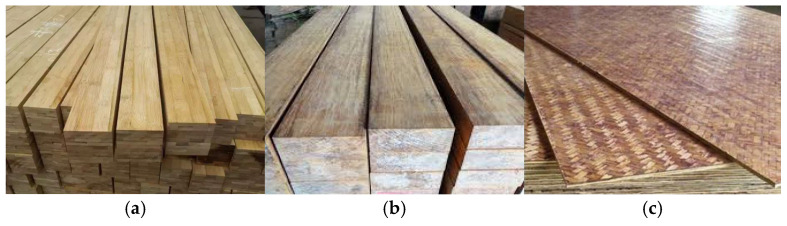
Engineered bamboo. (**a**) Laminated bamboo. (**b**) Bamboo scrimber. (**c**) Glubam.

Compared to timber, bamboo is another highly promising green and sustainable biomass resource. Amid the increasing pressure on forest resources, bamboo is becoming an important alternative to traditional timber due to its environmental benefits, abundance, and renewability. Using bamboo as a structural material and developing new bamboo–timber structures is expected to be a transformative breakthrough in the field of civil engineering. However, natural bamboo has defects such as anisotropy, dimensional inconsistency, susceptibility to cracking, and poor durability, which prevent it from meeting the basic mechanical performance requirements of modern building structures. As a result, engineered bamboo materials with excellent mechanical properties have been developed, such as laminated bamboo (Figure 7a), bamboo scrimber (Figure 7b), and glubam (Figure 7c).

**Figure 8:**
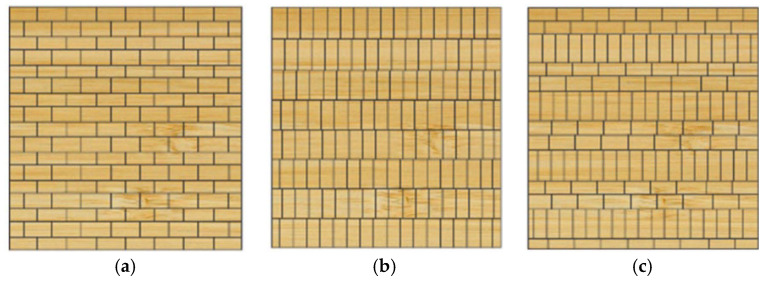
Laminated bamboo. (**a**) Horizontal. (**b**) Vertical. (**c**) Horizontal and vertical.

**Figure 9:**
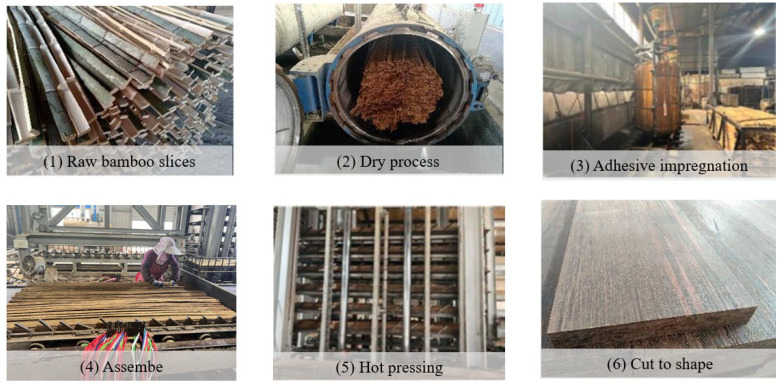
Production of bamboo scrimber.

**Figure 10:**
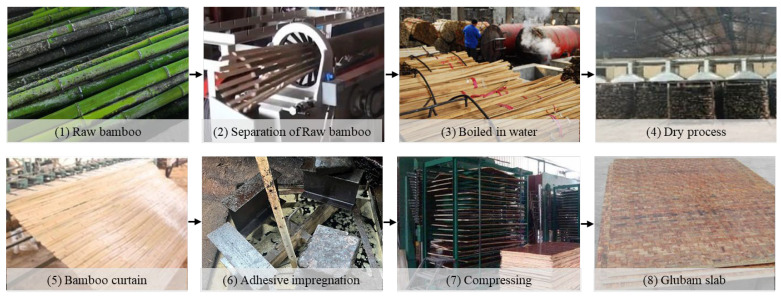
Production of glubam.

Integrated bamboo is a solid, gap-free engineered bamboo material, made by gluing bamboo strips and pressing them into structural components with fixed width and thickness. As shown in Figure 8, integrated bamboo can be classified into flat-press, side-press, and alternating flat-side laminated bamboo (cross-laminated) depending on the arrangement of the bamboo strips. Among these, side-press integrated bamboo exhibits better mechanical performance and is more commonly used in structural components, while flat-press and alternating flat-side laminated bamboo are more suitable for panel components. As shown in Figure 9, bamboo scrimber is a new type of bamboo material made from natural bamboo materials using hot-pressing or cold-pressing bonding processes. Specifically, the original bamboo is processed into bamboo strips or shredded into bamboo fibers, then oven-dried. The bamboo strips or fibers are then soaked in industrial glue and dried a second time. Afterward, the materials are placed in a pressure mold and pressed into shape using mechanical equipment, followed by heat curing under high temperature and pressure. Finally, the material is trimmed and shaped to form bamboo scrimber profiles. As shown in Figure 10, glubam is an orthotropic laminated bamboo material with bamboo fibers distributed in both longitudinal and transverse directions. The mechanical properties in the plane can be adjusted by changing the ratio of longitudinal (parallel to the grain) to transverse (perpendicular to the grain) bamboo fibers (bamboo mat ratio) to meet the strength requirements of different components [22,98].

With the advancement of bamboo industrial processes, many engineered bamboo constructions are gradually entering the public eye. Scholars have conducted in-depth studies on various performance indicators of engineered bamboo. In terms of material properties, experimental studies and theoretical analyses have been conducted on the basic mechanical properties of different types of engineered bamboo. In the research and application of bamboo bending members, bamboo beams or bamboo panels made from ordinary engineered bamboo still face issues such as low section stiffness, insufficient load-bearing capacity, and span capability. To address this, researchers have combined bamboo beams or panels with steel [25,26,27,28], FRP [99,100,101], and concrete materials [31,32,33,34,35], proposing various types of reinforced bamboo bending members, aiming to leverage the mechanical advantages of different materials to enhance the bending performance of bamboo members. At the structural level, shake table tests, overall overturning tests, and fire simulation tests have been conducted on lightweight laminated bamboo frame structures, yielding data on their dynamic characteristics and fire safety performance [102]. The research results indicate that the performance of engineered bamboo is similar to that of engineered timber. Based on further scientific research and engineering practice, it is expected to have broader and more challenging applications in multi-story structures (3–5 stories), large-span structures, composite structures, and even high-rise structures (greater than 6 stories). Additionally, the use of low-carbon building materials, such as bamboo, which represent a unique resource advantage in China, in large-span and high-rise applications, often translates into significant social and economic benefits.

## 4. CLBT Structure

**Figure 11:**
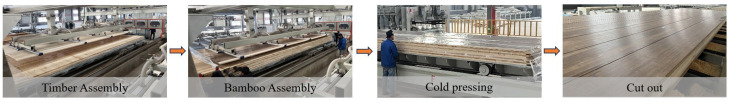
Process of CLBT production (Bamboo scrimber–SPF).

Building on the previous discussion, the combination of engineered bamboo and timber to produce a new composite structure, namely cross-laminated bamboo and timber (CLBT), theoretically not only offers superior mechanical performance but also fully utilizes bamboo resources, providing a more efficient, green, and sustainable alternative for modern construction. From a long-term perspective, CLBT represents not only an improvement and upgrade of traditional timber and bamboo processing technologies but also a dual contribution to modern architecture and the environment. The materials used in the production of CLBT components vary, such as glubam–timber, laminated bamboo–timber, bamboo scrimber–timber, and so on. The manufacturing process of CLBT is similar to that of CLT, and Figure 11 illustrates the production process of CLBT made from bamboo scrimber and SPF.

### 4.1. Beam Structure

**Figure 12:**
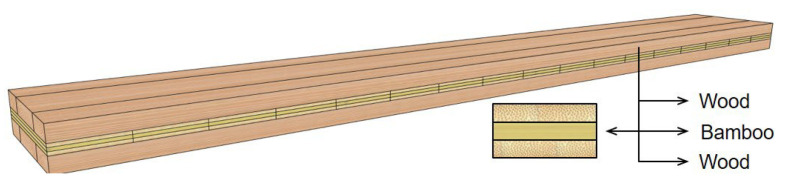
CLBT structure form developed by Wei et al. [105].

Due to the wide variety of engineered bamboo materials and the diverse forms of CLT structures, there are many types of CLBT. The following is a summary of the current CLBT structural forms. Xiao et al. [103] designed two types of five-layer CLBT beams with engineered bamboo on the surface and timber in the core. The timber used was imported SPF or domestic poplar, and the engineered bamboo used was either thick glubam or thin glubam. Li et al. [39] proposed two types of three-layer bamboo–timber composite CLT, prepared using bamboo mat–bamboo-panel plytimber, and iron-spruce: (1) the surface layer is iron-spruce and the inner layer is bamboo mat–bamboo-panel plytimber (WBW-CCLT); (2) the surface layer is bamboo mat–bamboo-panel plytimber and the inner layer is iron-spruce (BWB-CCLT). Dong et al. [104] proposed two types of multi-layer CLBT made from reconstituted bamboo and SPF: (1) the outer parallel layers are SPF boards with the internal transverse layers made of reconstituted bamboo boards (WBW-CLTB); (2) the outer parallel layers are thinner reconstituted bamboo boards and normal thickness SPF boards with internal transverse layers made of reconstituted bamboo boards (BWBWB-CLTB). As shown in Figure 12, Wei et al. [105] developed a new three-layer bamboo–timber composite CLT, where the outer layers are iron-spruce boards and the internal bamboo layers consist of three orthogonal layers of flattened bamboo boards. Zhang et al. [106] designed three sets of three-layer CLBT panels with flattened bamboo boards on the outer layers and domestic spruce on the inner layers. The difference among these three sets lies in the number of internal layers of the flattened bamboo boards. The bending test results of CLBT beams show that CLBT specimens made with engineered bamboo have significantly higher load-bearing capacity and stiffness compared to CLT specimens made with timber.

### 4.2. Column Structure

Research on the in-plane compressive performance of CLT has been relatively comprehensive, covering various types of short-column and long-column specimens. Currently, research on the in-plane compressive performance of CLBT is still in its early stages, with more studies focusing on CLBT short columns. Moreover, the structural form of CLBT short columns is similar to that of beams.

Li et al. [107] studied the in-plane off-axis compressive performance of two types of three-layer CLBT columns. The experimental results indicated that the main failure modes of the specimens were slender failure regions along or perpendicular to the off-axis angle, as well as delamination between layers. Li et al. [108] further investigated the in-plane compressive performance of CLBT specimens with off-axis angles of 0°, 45°, and 90°. The results showed that the distribution angle and combination method of timber and bamboo fibers had a significant impact on the compressive performance of the specimens. Additionally, the compressive performance exhibited significant angle dependence. Wei et al. [105] cut three-layer bamboo–timber composite CLT panels made of flattened bamboo and iron-spruce into short column specimens of three different heights and studied their in-plane compressive performance. The experimental results showed that at the same height, the compressive strength of the bamboo–timber composite CLT column specimens was similar to that of the iron-spruce CLT column specimens, but with a higher compressive elastic modulus. Due to the lower compressive strength of the timber layer in the bamboo–timber composite CLT columns, failure occurred. Furthermore, delamination failure occurred at the interface due to the mechanical property differences between the bamboo and timber layers. Fan et al. [42] investigated the mechanical performance of bamboo–timber composite columns with different slenderness ratios under axial compression. The three-layer composite columns had glubam as the outer layer and laminated veneer lumber (LVL) as the core layer. The study revealed that short columns, with a height of 800 mm, failed due to strength failure, whereas long columns, with heights of 1600 mm and 2400 mm, failed due to buckling. Strength failure could be classified into splitting failure, shear-compression failure, and local buckling failure.

### 4.3. Theoretical Basis of CLBT

Based on experimental research on CLBT, Xiao et al. [109] proposed an analytical model based on the Higher-Order Shear Deformation Theory (HOT) to further study the bending and axial compression performance of CLBT beams and columns. According to the Higher-Order Shear Deformation Theory, the displacement field along the height of the cross-section of a beam subjected to bending can be expressed as:

(1) $$u_{i}=u_{0}-y\theta_{i}+y^{2}\alpha_{i}+y^{3}\beta_{i}$$

(2) $$w=w_{0}$$

where: *u_i_* and *w* are the transverse displacement and longitudinal displacement of any point on the beam cross-section; *u*_0_ and *w*_0_ are the transverse displacement and longitudinal displacement at the point on the beam cross-section along the *x*-axis; *θ_i_* is the rotation angle of the beam cross-section due to bending deformation; *α_i_* and *β_i_* are the higher-order coefficients. In the proposed higher-order beam and column model, the governing equations are derived based on the principle of virtual work:

(3) $$\sum_{j=1}^{m}\int_{x}\int_{A_{j}}{\left\{\delta {\bar{\varepsilon }}_{j}\right\}}^{T}\left\{{\bar{\sigma }}_{j}\right\}dAdx-\delta W_{e}=0$$

(4) $$\left\{{\bar{\varepsilon }}_{j}\right\}=\left\{\begin{array}{c} \varepsilon_{j}\\ \gamma_{j} \end{array}\right\}=\left\{\begin{array}{c} \partial u_{j}/\partial x\\ \partial u_{j}/\partial y+\partial w/\partial x \end{array}\right\}$$

(5) $$\left\{{\bar{\sigma }}_{j}\right\}=\left[\begin{array}{cc} E_{j} & 0\\ 0 & G_{j} \end{array}\right]\left\{\begin{array}{c} \varepsilon_{j}\\ \gamma_{j} \end{array}\right\}=\left[D_{j}\right]\left\{\begin{array}{c} \varepsilon_{j}\\ \gamma_{j} \end{array}\right\}$$

When the multi-layer composite beam component is subjected to an external vertical load *q* (along the *y*-direction), the effect by the external force can be expressed as:

(6) $$\delta W_{e}=\int_{x}\delta wqdx$$

When the multi-layer composite column component is subjected to axial compression (load *P* along the *x*-direction), the effect the external force can be expressed as:

(7) $$\delta W_{e}=Pu_{0}(x=0)+Pu_{0}(x=l)+P\int_{x}\delta w\frac{d^{2}w}{dx^{2}}dx$$

Substituting Equations (1), (2), (6) and (7) into Equation (3) yields the final expression of the governing equation as:

(8) [K]{Δ}={Q}

(9) $$\{\Delta \}={\left[u_{0}\;\theta_{i}\;w_{0}\right]}^{T}$$

where [*K*] is the stiffness matrix; {Δ} is the deformation parameter vector; and {*Q*} is the external load vector.

### 4.4. Structure Design of CLBT

Similarly to CLT structures, CLBT has the potential for application in multi-story building structures. By referring to and integrating the application and practices of multi-story CLT structures internationally, further research and demonstration projects should be carried out on the application of CLBT structures in multi-story buildings, to promote the use of CLBT materials in structural engineering.

The development of modern science and technology has gradually transformed traditional architectural design thinking, which relied on points and lines as basic construction units, into a nonlinear design approach based on planes as the fundamental building blocks. For CLBT, unlike traditional frame timber structures, where beams and columns are the primary structural components, the basic structural units of CLBT materials are panels. Therefore, when designing buildings, the design must focus on planes (or curved surfaces) as the basic building units.

CLBT demonstration buildings can adopt a plate shear wall structure, where both the walls and floors use CLBT panels. The box-shaped structural system formed by the walls and floors provides vertical and lateral load-bearing capacity. CLBT shear wall structures can enable prefabrication of components, followed by on-site assembly. Prefabricated components improve construction efficiency. This approach requires applying an assembly-based modular design concept, where the module serves as the basic unit of the building, and the form and design of these units are key to the success of the construction project. Modular CLBT structures can also be combined with other materials (concrete, steel, and so on.), fully utilizing the modular and component-based characteristics of CLBT structures to create flexible and adaptable building spaces, meeting ever-changing demands and unforeseen events.

### 4.5. Critical Discussion and Research Synthesis

A critical evaluation of the current research landscape reveals that while CLBT exhibits promising mechanical properties, its transition from laboratory research to widespread structural application is constrained by several fundamental and interconnected challenges. These gaps represent the most urgent priorities for future investigation.

- Fire Performance: A Critical Unresolved Challenge

The fire resistance of CLBT remains a significant and unquantified risk. The potential for differential thermal expansion and delamination at the bamboo–timber interface under high temperatures could compromise structural integrity by accelerating charring. Research must urgently prioritize quantifying charring rates, understanding delamination mechanisms, and developing specific fire protection strategies for this hybrid system.

- Long-Term Durability and Environmental Behavior

The performance of CLBT under sustained loading (creep) and in variable hygrothermal conditions is largely unknown. The differing moisture responses of bamboo and timber may induce internal stresses, leading to adhesive degradation or dimensional instability over time. Systematic studies using accelerated aging protocols and long-term real-world exposure are essential to predict service life and establish reliable design parameters for durability.

- Connection Design and System-Level Integration

The development of CLBT has predominantly focused on member-level performance, creating a critical gap in understanding system-level behavior, particularly at connections. The higher density and hardness of bamboo layers alter the embedment behavior and failure modes of connectors such as screws and bolts. There is an urgent need to develop, test, and systematize efficient, ductile, and reliable connection details for panel-to-panel and wall-to-floor assemblies, especially for seismic and dynamic loading scenarios.

- Standardization and Sustainability Validation

The absence of standardized testing protocols, grading rules, and design guidelines constitutes a primary barrier to commercial adoption and regulatory acceptance. Furthermore, while environmental benefits are frequently cited, comprehensive and quantitative Life Cycle Assessment (LCA) studies are lacking to validate the sustainability claims of CLBT compared to conventional materials.

A comparative summary of the key characteristics between conventional CLT and emerging CLBT is provided in Table 1. This synthesis highlights the comparative advantages and challenges of CLBT, underscoring its potential as a superior sustainable building material while also clarifying the critical research and development efforts required for its standardization and widespread adoption.

In summary, addressing these four prioritized research areas—fire safety, long-term durability, system connections, and standardization—is imperative to bridge the gap between experimental validation and the safe, reliable, and codified use of CLBT in structural engineering.

## 5. Conclusions and Outlook

This paper has provided a comprehensive review of the research progress on Cross-Laminated Timber (CLT) and the emerging field of Cross-Laminated Bamboo and Timber (CLBT). The development of CLT, from a novel engineered wood product to a material underpinning modern sustainable construction, offers a robust foundation for the development of CLBT. The primary conclusions and forward-looking perspectives are summarized as follows:

(1) CLT as a Mature Building Material: CLT technology has reached a significant level of maturity, evidenced by its successful application in multi-story and high-rise buildings globally. Its mechanical performance, including bending, shear, and compressive strength, is well understood. Research has demonstrated that strategies such as using hybrid species, optimizing layup configurations (e.g., 45° orientation), and adjusting layer parameters can effectively enhance its rolling shear resistance and overall structural efficiency. Furthermore, CLT exhibits remarkable resilience under extreme conditions, characterized by excellent integrity during seismic events and predictable charring behavior under fire exposure, especially when integrated with modern connection systems and energy-dissipating devices.
(2) CLBT as a High-Performance Successor: Building on the CLT concept, CLBT emerges as a promising next-generation composite that synergizes the high strength and stiffness of engineered bamboo with the favorable processing attributes of timber. Experimental investigations into various CLBT beam and column configurations consistently report superior load-bearing capacity and stiffness compared to conventional softwood CLT. Failure typically initiates in the timber layers or adhesive interfaces, underscoring the effective role of the bamboo reinforcement. Analytical models, particularly those based on higher-order shear deformation theory, provide a valuable foundation for understanding and predicting the complex mechanical behavior of CLBT under various loading conditions.
(3) Future Pathways Guided by Critical Research Gaps: While the foundational research is promising, the pathway to standardization and widespread adoption of CLBT requires targeted efforts to address critical knowledge gaps identified in this review. Future work should be strategically prioritized in the following domains to bridge the gap between laboratory research and full-scale structural application:
  ● Material Performance and Durability: Advancing the development of hybrid bamboo–timber combinations and dedicated adhesive systems is crucial. A paramount focus must be placed on understanding the long-term performance, including creep behavior and durability under fluctuating hygrothermal conditions, to ensure structural integrity over the lifecycle of buildings.
  ● Structural System Safety and Reliability: Urgent research is needed to establish the fire resistance rating of CLBT assemblies and to develop robust, ductile connection details capable of performing under seismic loads. The behavior of CLBT at the structural system level, rather than just the component level, requires comprehensive investigation through full-scale testing and advanced numerical modeling.
  ● Standardization and Sustainability Validation: The transition to commercial application necessitates the development of standardized testing methods, grading rules, and design guidelines specifically tailored for CLBT. Concurrently, comprehensive Life Cycle Assessment (LCA) studies are essential to quantitatively validate and communicate the environmental benefits of CLBT, providing a solid basis for its recognition as a sustainable construction material.

In summary, CLBT represents a significant innovation in the pursuit of sustainable and high-performance bio-based construction materials. By addressing these prioritized research challenges, the scientific and engineering community can unlock the full potential of CLBT, facilitating its integration into building codes and its application in the next generation of green buildings.

## Figures and Tables

**Table 1:** Comparative summary between Cross-Laminated Timber (CLT) and Cross-Laminated Bamboo and Timber (CLBT).

	Aspect	Cross-Laminated Timber (CLT)	Cross-Laminated Bamboo and Timber (CLBT)
Mechanical Performance	Rolling shear strength	Moderate	Significantly enhanced (due to high shear modulus of bamboo)
	Bending stiffness/strength	Good, but limited by cross-layer properties	Superior (high-strength bamboo layers improve load-bearing capacity and stiffness)
	Failure mode	Rolling shear in cross-layers; interlaminar shear failure	Failure initiates in timber layers or at adhesively bonded interfaces; improved ductility
Sustainability	Raw material source	Primarily relies on softwood (e.g., spruce, fir)	Diversified (utilizes fast-growing bamboo and a broader range of timber species)
	Biomass utilization	Good	Excellent (promotes high-value utilization of rapidly renewable bamboo resources)
	Carbon footprint	Low (carbon-sequestering)	Potentially lower (bamboo’s extremely short growth cycle enhances carbon sequestration efficiency)
Challenges & Maturity	Standardization & design codes	Well-established	Under development (Lack of specific grading rules, test standards, and design guidelines)
	Fire resistance	Relatively well-understood; charring rates documented	A critical unknown (Performance of bamboo–timber interface under fire; delamination risks need urgent investigation)
	Long-term durability & creep	Well-studied and characterized	Lacking research (Differential hygrothermal responses may induce internal stresses affecting long-term integrity)
	Connection design	Mature, with numerous validated solutions	Requires new research (Higher density/hardness of bamboo layers alters connector embedment behavior and failure modes)
	Engineering application scale	Widespread (Used in multi-story and high-rise buildings)	Predominantly lab-scale research and demonstrations (Distance from large-scale commercial application)

## Data Availability

No new data were created or analyzed in this study. Data sharing is not applicable to this article.
